# Quality of Recovery and Innate Immune Homeostasis in Patients Undergoing Low-pressure Versus Standard-pressure Pneumoperitoneum During Laparoscopic Colorectal Surgery (RECOVER): A Randomized Controlled Trial

**DOI:** 10.1097/SLA.0000000000005491

**Published:** 2022-07-13

**Authors:** Kim I. Albers, Fatih Polat, Leonie Helder, Ivo F. Panhuizen, Marc M.J. Snoeck, S. (Bas) W. Polle, Hilbert de Vries, Esther M. Dias, Gerrit D. Slooter, Hans D. de Boer, Oscar Diaz-Cambronero, Guido Mazzinari, Gert-Jan Scheffer, Christiaan Keijzer, Michiel C. Warlé, RECOVER Study Collaborators

**Affiliations:** * Department of Anesthesiology, Radboudumc, Nijmegen, The Netherlands; † Department of Surgery, Radboudumc, Nijmegen, The Netherlands; ‡ Department of Surgery, Canisius Wilhelmina Hospital, Nijmegen, The Netherlands; § Department of Anesthesiology, Canisius Wilhelmina Hospital, Nijmegen, The Netherlands; ∥ Department of Anesthesiology, Maxima Medical Center, Veldhoven, The Netherlands; ¶ Department of Surgery, Maxima Medical Center, Veldhoven, The Netherlands; # Department of Anesthesiology, Pain Medicine and Procedural Sedation and Analgesia, Martini General Hospital, Groningen, The Netherlands; ** Department of Anesthesiology, La Fe University and Polytechnic Hospital, Valencia, Spain

**Keywords:** laparoscopy, laparoscopic surgery, low pressure pneumoperitoneum, intra-abdominal pressure, deep neuromuscular blockade, QoR-40, DAMPs, innate immunity, postoperative infections

## Abstract

To study the effects of intra-abdominal pressure on the quality of recovery and innate cytokine production capacity after laparoscopic colorectal surgery within the enhanced recovery after surgery program.

**Background:**

There is increasing evidence for the safety and advantages of low-pressure pneumoperitoneum facilitated by deep neuromuscular blockade (NMB). Nonetheless, there is a weak understanding of the relationship between clinical outcomes, surgical injury, postoperative immune dysfunction, and infectious complications.

**Methods:**

Randomized controlled trial of 178 patients treated at standard-pressure pneumoperitoneum (12 mm Hg) with moderate NMB (train-of-four 1–2) or low pressure (8 mm Hg) facilitated by deep NMB (posttetanic count 1–2). The primary outcome was the quality of recovery (Quality of Recovery 40 questionnaire) on a postoperative day 1 (POD1). The primary outcome of the immune substudy (n=100) was ex vivo tumor necrosis factor α production capacity upon endotoxin stimulation on POD1.

**Results:**

Quality of Recovery 40 score on POD1 was significantly higher at 167 versus 159 [mean difference (MD): 8.3 points; 95% confidence interval (CI): 2.5, 14.1; *P*=0.005] and the decline in cytokine production capacity was significantly less for tumor necrosis factor α and interleukin-6 (MD: −172 pg/mL; 95% CI: −316, −27; *P*=0.021 and MD: −1282 pg/mL; 95% CI: −2505, −59; *P*=0.040, respectively) for patients operated at low pressure. Low pressure was associated with reduced surgical site hypoxia and inflammation markers and circulating damage-associated molecular patterns, with a less impaired early postoperative ex vivo cytokine production capacity. At low pressure, patients reported lower acute pain scores and developed significantly less 30-day infectious complications.

**Conclusions:**

Low intra-abdominal pressure during laparoscopic colorectal surgery is safe, improves the postoperative quality of recovery and preserves innate immune homeostasis, and forms a valuable addition to future enhanced recovery after surgery programs.

Laparoscopic surgery is one of the key components of the enhanced recovery after surgery (ERAS) program for elective colorectal surgery, advocated for its advantages regarding recovery and reduction in complications compared with open surgery.^1^,^2^ While the consensus guidelines of the European association for endoscopic surgery advise to use the lowest possible intra-abdominal pressure (IAP) with an adequate view of the surgical field,^3^ the 2018 ERAS® Society guideline for Perioperative Care in Elective Colorectal Surgery states that evidence for reducing IAP below 10 to 12 mm Hg is low. Since then, there is increasing evidence that low-pressure pneumoperitoneum (LPP) facilitated by deep neuromuscular blockade (NMB) is both safe and feasible while offering physiological and clinical advantages. Diaz-Cambronero et al^4^ used a blinded individualized strategy to titrate IAP and found that 78% of colorectal surgeries could safely be completed at 8 mm Hg. Kim et al^5^ reported surgical conditions were maintained at LPP with deep NMB and resulted in less postoperative pain and a faster bowel recovery. The PAROS trial from Celarier et al^6^ showed a shorter length of hospital stay, lower pain scores, and less opioid consumption after colectomy at low IAP. In most trials investigating IAP, low pressure is facilitated by deep NMB as evidence supports it improves surgical conditions and therefore safety of low pressure during laparoscopy.^7^ When comparing to standard pressure and NMB, there are essentially 2 components to the intervention: IAP and NMB. Meta-analysis on the effects of deep NMB performed by our group^7^ and recently updated by Raval et al^8^ reveal a small difference in favor of deep NMB regarding pain scores at the postanesthesia care unit (PACU), which is driven by one low-pressure study. Therefore, deep NMB may slightly reduce pain scores at the PACU, but evidence shows no benefit of NMB on pain scores and recovery after 24 hours. LPP of 8 mm Hg is associated with a significantly improved perfusion of the parietal peritoneum compared with standard-pressure pneumoperitoneum (SPP) of 12 mm Hg.^9^ For prolonged exposure during laparoscopic surgery, lowering IAP to 8 mm Hg may reduce hypoxia-reperfusion injury and thereby decrease the amount of circulating damage-associated molecular patterns (DAMPs). DAMPs are intracellular molecules that become exposed when cells are damaged or can be secreted by cells in danger. They act as ligands for toll-like receptors on innate immune cells and lead to immune suppression.^10^,^11^ We strive to elucidate the effects of IAP-related surgical injury on the cytokine production capacity of innate immune cells. We hypothesize that compared with SPP, LPP will improve the quality of recovery and preserve innate immune homeostasis after colorectal laparoscopy within the ERAS program.

## METHODS

The RECOVER study was a multicenter double-blinded randomized controlled trial performed at 3 general teaching hospitals in The Netherlands between October 2018 and March 2021, assessing the effects of LPP facilitated by deep NMB versus SPP and moderate NMB on quality of recovery in patients undergoing colorectal laparoscopic surgery. The complete methods of the RECOVER study (clinicaltrials.gov NCT03608436) have been described in the published study protocol.^12^ In addition, an immunological substudy (RECOVER PLUS, clinicaltrials.gov NCT03572413) was performed in the first 100 patients enrolled at the Canisius Wilhelmina Hospital. Both protocols were approved by the Medical Research Ethics Committee “CMO region Arnhem-Nijmegen” and the competent authority (Central Committee on Research Involving Human Subjects). All patients provided informed consent for participation in the trial.

### Treatment and Clinical Outcomes

Patients were randomized in a 1:1 fashion to LPP (8 mm Hg) with deep NMB defined as a posttetanic count (PTC) of 1–2, or SPP (12 mm Hg) with moderate NMB defined as a train-of-four count of 1–2. Randomization was stratified for center and robot assistance. The surgeon was blinded to the study arm and level of IAP and rated the quality of the surgical field on the Leiden Surgical Rating Scale (L-SRS) every 15 minutes. In case of inadequate surgical conditions (L-SRS ≤3 of 5 at any time during the surgery), IAP was increased with 2 to 10 mm Hg and a maximum of 12 mm Hg for LPP or 14 mm Hg and a maximum of 16 mm Hg for SPP. The primary outcome was the patient-reported quality of recovery on a postoperative day (POD), measured with the Dutch version of the validated Quality of Recovery 40 (QoR-40) questionnaire.^13^ Adherence to 29 preoperative, intraoperative, and postoperative key elements of the ERAS Society guideline was scored for all patients.^1^,^14^ Secondary outcome measures were quality of the surgical field (mean L-SRS score), blood loss, intraoperative complications classified by the ClassIntra classification,^15^ pain, nausea, use of analgesics and antiemetics, QoR-40 on POD3 and POD7, length of hospital stay, time to reach discharge criteria, 30-day postoperative complications classified by the Clavien-Dindo^16^ classification, health-related quality of life (HRQOL) 3 months after surgery measured by the Dutch version of the Research and Development-36 (RAND-36)^17^ questionnaire and chronic pain measured with the Dutch version of the McGill Pain Questionnaire (MPQ)^18^ 3 months after surgery. Except for the anesthesiologist (who only assessed peroperative anesthesiologic complications) all outcome assessors were blinded to the study arm.

### RECOVER PLUS

In patients enrolled in the substudy, blood was drawn by venipuncture before surgery, at the end of the surgery, on POD1 and POD3 when still admitted at that time. Whole blood ex vivo cytokine production capacity upon endotoxin stimulation, plasma DAMP levels, and plasma cytokine concentrations were quantified as previously described, for detailed methodology we refer to these publications.^8^,^9^ The primary outcome of the immune substudy was the change in ex vivo tumor necrosis factor α (TNFα) production capacity on POD1 upon whole blood endotoxin stimulation. Secondary (explorative) outcomes were change in ex vivo production capacity of interleukin (IL)-6, IL-1β, and IL-10, plasma DAMPS (HSP70, HMGB1, nDNA, and mtDNA), plasma cytokines (TNFα, IL-10, and IL-6) and local peritoneal tissue hypoxia and inflammation markers [hypoxia-inducible factor 1α (HIF1α), vascular endothelial growth factor (VEGF), TNFα, IL-6, and IL-1β]. For the endotoxin stimulation, 0.5 mL of lithium heparin anticoagulated whole blood was added to preprepared tubes with 2 mL culture medium (negative control) and 2 mL culture medium supplemented with 12.5 ng/mL *Escherichia coli* lipopolysaccharide (serotype O55:B5 Sigma Aldrich, St Louis, MO) in a biosafety cabinet, resulting in a final concentration of 10 ng/mL. Tubes were prepared in one batch, stored at −80°C, and thawed shortly before use. After adding the blood, the tubes were cultured at 37°C for 24 hours, then centrifuged for 5 minutes at 1500 rpm and the supernatants were stored at −80°C until analysis. Supernatant cytokine levels were measured by enzyme-linked immunosorbent assay (R&D Systems, Minneapolis, MN) according to the manufacturer’s instructions. Plasma DAMP concentrations were determined from doubly centrifuged EDTA anticoagulated blood. DNA was isolated with the QIAamp DNA Blood Midi Kit (Qiagen, Valencia, CA) and levels of nDNA and mtDNA were determined by quantitative polymerase chain reaction (PCR) on a CFX96 Real-Time PCR Detection System (Bio-Rad Laboratories, Hercules, CA) and expressed as fold change relative to preoperative values of the same patient using the formula: 2^Δ*C*t^. Concentrations of HSP70 (R&D Systems) and HMGB1 (IBL International GmbH, Hamburg, Germany) were measured batchwise by ELISA according to the manufacturer’s instructions. Plasma concentrations of TNFα, IL-10, and IL-6 were determined batchwise using a simultaneous Luminex assay (Milliplex; Millipore, Billerica, MA) according to the manufacturer’s instructions.

For the first 20 substudy patients, peritoneal biopsies (0.5–1 by 0.5–1 cm) were taken right after abdominal insufflation and at the end of surgery. Biopsies were collected in RNAlater tissue protect tubes (Qiagen, Germantown, MD) and stored at −80°C until analysis. mRNA was extracted with the Qiagen RNA extraction kit, and HIF1α, VEGF, TNFα, IL-1β, and IL-6 levels were determined by quantitative PCR.

### Statistical Analysis

To achieve 80% power to detect a mean clinically important difference of 6.3^19^ on the QoR-40 score (SD=15,^17^ range: 40–200) with an α of 5%, a sample size of 89 per group (178 total) was required. The published protocol describes 204 participants because of an estimated 15% conversion rate to open surgery. As the actual conversion rate was much lower, patients were enrolled until both groups reached 89 participants for the final analysis. A sample size of 48 patients per group was needed to provide 90% power to detect a 150 pg/mL difference in TNFα release from baseline to POD1 upon endotoxin stimulation (α of 5%) with an estimated SD of 225 pg/mL.^11^

All statistical analyses were performed using Statistical Package for the Social Sciences (IBM SPSS Statistics version 27; IBM Corp., Armonk, NY). Continuous data were presented as mean±SD and categorical data were presented as a number with a percentage. We did not perform data imputation for missing data. For the primary outcome analysis, analysis of covariance was used to compare the QoR-40 score on POD1 between LPP and SPP, controlled for covariates age, sex, body mass index, and American Society of Anesthesiology classification. For secondary outcome variables, a Student *t* test was used to compare normally distributed continuous variables and the χ^2^ test for categorical variables. A *P* value <0.05 was considered statistically significant. For the correlation matrix, Pearson *r* was calculated for continuous variables that were normally distributed, Spearman ρ for skewed variables.

## RESULTS

**FIGURE 1:**
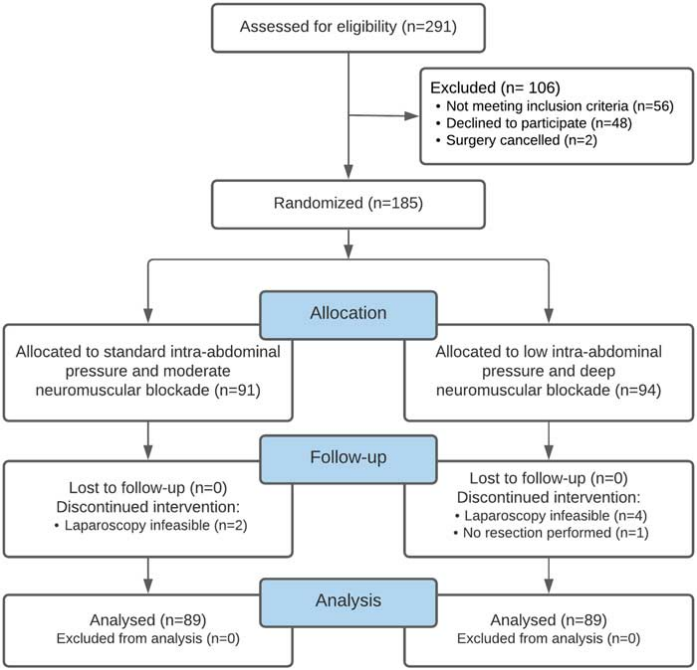
CONSORT flowchart.

A CONSORT flowchart of screening and treatment allocation is shown in Figure 1, 185 patients were randomized, 7 patients were excluded because laparoscopy was infeasible (n=6, 3.2%) or no colonic resection was performed (n=1, 0.5%), 178 patients were included in the final analysis. For all excluded cases, the unfeasibility of laparoscopy was due to patient or tumor characteristics unrelated to IAP or NMB. Baseline characteristics were similar between groups as listed in Table 1.

**TABLE 1:** Baseline Characteristics

	Main Study (N=178)			Substudy (N=100)		
	Standard Pressure and Moderate NMB (n=89)	Low Pressure and Deep NMB (n=89)	*P*	Standard Pressure and Moderate NMB (n=50)	Low Pressure and Deep NMB (n=50)	*P*
Hospital (CWZ/MMC/Martini)	74/9/6 (83/10/7)	73/8/8 (82/9/9)	0.704	50/0/0 (100/0/0)	50/0/0 (100/0/0)	1.000
Sex (male/female)	57/32 (64/36)	57/32 (64/36)	1.000	29/21 (58/42)	35/15 (70/30)	0.215
Age (y)	68.9±9.2	68.5±9.5	0.816	69.0±9.5	68.7±9.2	0.837
Height (cm)	175±9	174±10	0.551	174±10	174±10	0.948
Weight (kg)	83±16	79 ±15	0.106	82±18	80±15	0.530
BMI (kg/m^2^)	27.3±4.8	26.2±4.0	0.110	26.9±5.0	26.2±3.6	0.430
ASA (I/II/III)	22/48/19 (25/54/21)	19/56/14 (21/63/16)	0.817	12/28/10 (24/56/20)	10/32/8 (20/64/16)	1.000
Laparoscopic/robot-assisted	44/45 (49/51)	38/51 (43/57)	0.370	24/26 (48/52)	21/29 (42/58)	0.551
Type of surgery			0.427			0.668
Right hemicolectomy	29 (33)	34 (38)		16 (32)	14 (28)	
Sigmoid resection	34 (38)	25 (28)		19 (38)	20 (40)	
Low anterior resection/TME/PME	16 (18)	19 (21)		9 (18)	9 (18)	
Left hemicolectomy	6 (7)	8 (9)		3 (6)	5 (10)	
Ileocecal resection	2 (2)	2 (2)		2 (4)	1 (2)	
Right hemicolectomy*+*sigmoid resection	1 (1)	1 (1)		1 (2)	1 (2)	
Subtotal colectomy	1 (1)					
Surgery indication			0.254			0.361
Malignancy	78 (88)	80 (90)		43 (86)	44 (88)	
Benign pathology (adenoma, volvulus)	5 (6)	5 (6)		4 (8)	3 (6)	
Inflammatory (Crohn’s disease, diverticulitis)	6 (7)	4 (4)		3 (6)	3 (6)	

Presented values are absolute n (%) or mean±SD.

ASA indicates American Society of Anesthesiologists classification; BMI, body mass index; CWZ, Canisius Wilhelmina Hospital; MMC, Maxima Medical Centre; PME, partial mesorectal excision; TME, total mesorectal excision.

### Primary Outcome

**FIGURE 2:**
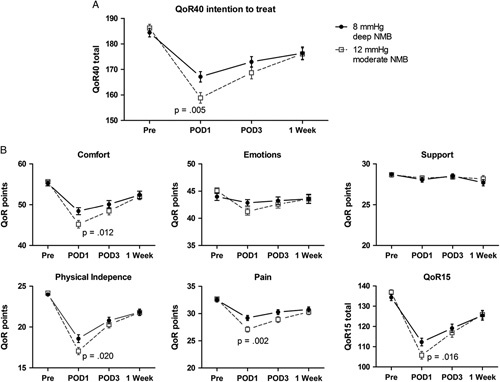
QoR-40 overall and per domain. Total QoR-40 score analyzed by intention to treat (n=89 in both groups) (A) with separate domains and QoR-15 in (B).

The mean quality of recovery, QoR-40, on POD1 was significantly better for LPP and deep NMB (mean: 167) compared with SPP and moderate NMB (mean: 159) [mean difference (MD): 8.3; 95% confidence interval (CI): 2.5, 14.1; *P*=0.005]. The covariates age and sex were significantly related to QoR-40 on POD1 (*F*_1,169_=5.91, *P*=0.016 and *F*_1,169_=4.30, *P*=0.040, respectively), whereas body mass index and American Society of Anesthesiology classification were not. The effect of low pressure on QoR-40 remained statistically significant after controlling for these covariates (*F*_1,169_=7.92, *P*=0.005). Baseline mean QoR-40 was 184 for LPP versus 186 for SPP (MD: 1.8; 95% CI: −6.2, 2.6; *P*=0.420). Figure 2A shows the total QoR-40 scores by intention-to-treat analysis (n=89 in both groups), Figure 2B shows the separate domains and illustrates benefits in pain, comfort, and physical independence. A significant difference on POD1 was also seen on the QoR-15 (range: 0−150) with a mean of 112 for LPP versus 106 for SPP (MD: 6.5; 95% CI: 1.2–11.8; *P*=0.016).

### Secondary Outcomes

#### Intraoperative and Postoperative Clinical Outcomes

Intraoperative outcomes are presented in Table 2. Mean IAP for patients randomized to LPP was 8.7 mm Hg, compared with 12.4 mm Hg at SPP. Requested increases in IAP were generally at the beginning of surgery, 74% within the first 15 minutes. No statistically significant differences were found between groups for the duration of surgery, quality of the surgical field, intraoperative complications, or blood loss. There were no statistically significant differences in mean propofol (8.7±1.9 mg/kg/h), remifentanil (11.7±4.2 mcg/kg/h), lidocaine (1.8±0.6 mg/kg/h), esketamine (0.22±.08 mg/kg), morphine (0.1±0.03 mg/kg), or vasopressor dose in norepinephrine equivalents^20^ (0.0043±.01 μg/kg/min). Table 3 illustrates significantly lower postoperative pain scores and nausea for LPP. Last, patients in the LPP group developed significantly less infectious complications compared with patients in the SPP group [n=6 (7%) vs n=15 (17%), odds ratio=2.8; 95% CI: 1.03, 7.6; *P*=0.037, Table 4].

**TABLE 2:** Intraoperative Outcomes

	Standard Pressure and Moderate NMB (N=89)	Low Pressure and Deep NMB (N=89)	*P*
Duration of surgery (min)	157±49	161±52	0.592
Duration of pneumoperitoneum (min)	122±46	128±53	0.464
IAP			
Mean (mm Hg)	12.4	8.7	
Completed at initial set pressure	76 (85)	67 (75)	
+2 mm Hg	9 (10)	12 (14)	
+4 mm Hg	4 (5)	10 (11)	
NMB			
Mean (TOF/PTC)	TOF=1.9	PTC=1.4	<0.001
% of measurements exactly on target	67	73	
Rocuronium total (mg)	89±29	157±50	<0.001
Rocuronium (mg/kg/h)	0.44±0.13	0.79±0.27	
L-SRS	4.7±0.5	4.6±0.5	0.071
Ileostomy	4	5	0.734
Estimated blood loss (mL)	42±94	42±85	0.980
Intraoperative complications*	13 (15)	10 (11)	0.383
Venous bleeding	4	3	
Arterial bleeding	4	0	
Traction/cauterization injury	3	5	
Arrhythmia	2	0	
Other	0	2	

Presented values are absolute n (%) or mean±SD.

* All ClassIntra grade II (grade I was not recorded, grade III or higher did not occur).

TOF indicates train-of-four.

**TABLE 3:** Postoperative Outcomes

	Standard Pressure and Moderate NMB (N=89)	Low Pressure and Deep NMB (N=89)	*P*
Pain at rest (0–10)			
PACU	5.8±2.5	4.7±2.6	**0.004**
POD1	3.3±1.8	2.7±1.6	**0.016**
POD3	2.2±1.8	1.5±1.4	**0.015**
Pain upon movement (0–10)			
PACU	6.2±2.2	5.1±2.3	**<0.001**
POD1	4.8±1.9	4.5±2.0	0.254
POD3	3.9±1.6	3.1±1.8	**0.010**
Pain acceptable (yes/no)			
PACU	55/33 (63/38)	67/22 (75/25)	0.067
POD1	79/10 (89/11)	84/5 (94/6)	0.179
POD3	49/5 (91/9)	58/2 (97/3)	0.203
Referred shoulder pain (y/n)			
PACU	2/87 (2/98)	2/87 (2/98)	0.991
POD1	12/77 (13/87)	8/81 (9/91)	0.345
POD3	3/51 (6/94)	3/57 (5/95)	0.896
Nausea (0–10)			
PACU	1.3±2.3	0.7±1.5	**0.044**
POD1	1.5±2.3	0.6±1.4	**0.002**
POD3	1.3±2.4	0.8±1.6	0.233
Opioid consumption (morphine milligram equivalent)			
PACU	6.9±5.1	5.7±5.2	0.135
POD1	23.7±16.4	19.9±14.7	0.102
POD3	5.8±8.7	4.4±7.2	0.347
Hospital stay (d)			
Median	3	3	0.880
ERAS (%)			
Adherence	82	82	0.772
Discharge criteria (out of 5)			
POD1	2.6±1.4	3.0±1.4	0.054
POD3	4.2±1.3	4.3±1.1	0.450
HRQOL (RAND-36)			
Preoperative	76.7±12.1	72.5±16.4	0.077
After 3 mo	76.9±15.3	76.4±16.5	0.858
Δpreoperative—3 mo	0.1±10.6	3.9±12.4	**0.047**
MPQ			
NWC-T preoperative	1.66±3.3	2.08±3.5	0.409
NWC-T 3 mo	1.63±3.8	0.79±2.0	**0.049**
ΔNWC-T	0.03±3.7	1.29±3.1	**0.028**
PRI-T preoperative	2.66±6.0	3.53±6.1	0.170
PRI-T 3 mo	2.76±7.0	1.22±3.2	**0.045**
ΔPRI-T	−0.01±6.8	2.31±4.6	**0.019**

The statistically significant *P*-values are in bold.

Presented values are absolute n (%) or mean±SD.

**TABLE 4:** Postoperative Complications

	Standard Pressure and Moderate NMB (N=89)	Clavien-Dindo	Low Pressure and Deep NMB (N=89)	Clavien-Dindo	*P*
30-d infectious complications [n (%)]	15 (17)		6 (7)		**0.037**
	Anastomotic leak	IVb	Anastomotic leak	IIIb	
	Pneumonia	IVb	Infected hematoma	IIIa	
	Anastomotic leak	IIIb	2× Wound infection	II	
	Wound abscess	IIIb	Infected hematoma	II	
	Infected hematoma	IIIa	Abdominal infection	II	
	Fever with abdominal focus	IIIa			
	2× urinary tract infection	II			
	2× pneumonia	II			
	2× infected hematoma	II			
	Intra-abdominal abscess	II			
	Wound abscess	II			
	Cholecystitis	II			
30-d other complications [n (%)]	15 (17)		16 (18)		0.843
	Respiratory insufficiency	IVb	Aspiration pneumonia	V	
	Fascial dehiscence	IIIb	Fascial dehiscence	IIIb	
	GI bleeding	IIIa	Cicatricial hernia	IIIb	
	2× GI bleeding	II	Rotated ileostomy	IIIb	
	7× Ileus/gastroparesis	II	Abdominal hematoma	IIIb	
	Pulmonary emboli	II	2× GI bleeding	IIIa	
	Hypoxia due to atelectasis	II	8× ileus/gastroparesis	II	
	Atrial fibrillation de novo	II	Abdominal hematoma	II	

The statistically significant *P*-values are in bold.

GI indicates gastrointestinal.

#### Immune Outcomes, Surgical Injury, and Pain

**FIGURE 3:**
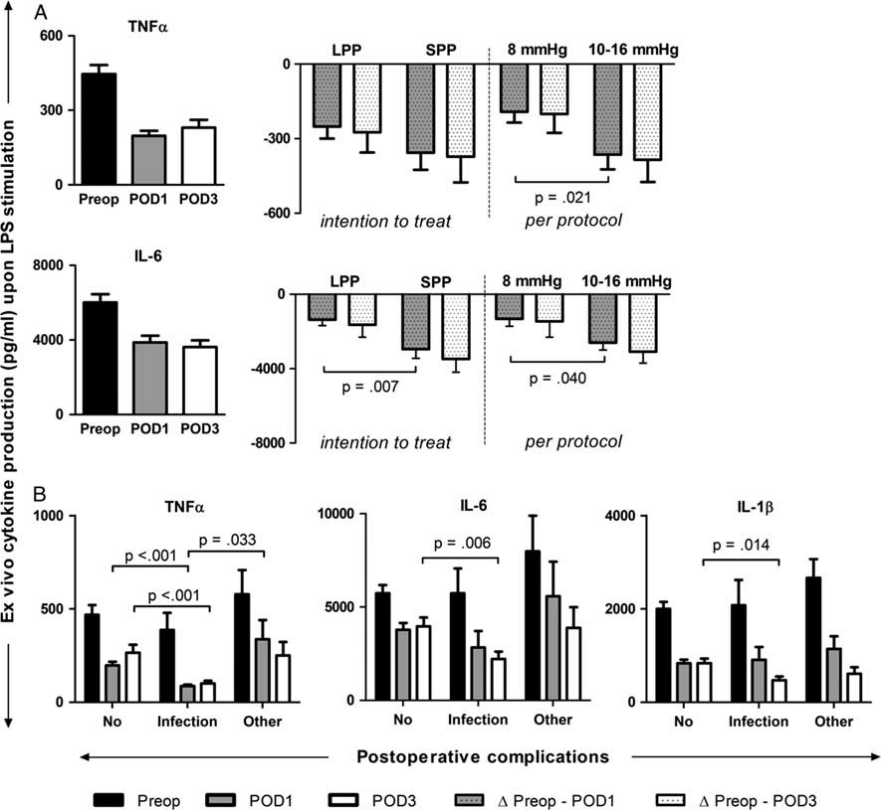
A, Ex vivo cytokine production capacity upon whole blood endotoxin stimulation and the effects of LPP for LPP and SPP (intention to treat, n=50 vs n=49) and 8 and 10 to 16 mm Hg (per-protocol, n=35 vs n=64). B, Ex vivo cytokine production (TNFα, IL-6, and IL-1β) for patients with no complications (n=73), infectious complications (n=15), and other complications (n=12). Data are represented as mean±SEM. Preop indicates preoperative.

Ex vivo production capacity of TNFα and IL-6 was strongly decreased on POD1 and POD3 compared with the preoperative state (Fig. 3A; from 468±427 to 198±197 to 231±254 pg/mL for TNFα and from 6009±4415 to 3865±3624 to 3614±3022 for IL-6). This is also seen for IL-1β (from 2091±1453 pg/mL before surgery to 882±767 pg/mL on POD1 and 719±587 pg/mL on POD3) and IL-10 production (from 151±261 to 104±183 to 88±163 pg/mL). The decrease in production capacity from preoperative to POD1 is significantly smaller at LPP compared with SPP for TNFα (193±249 pg/mL for LPP vs 364±477 pg/mL for SPP, MD: 172 pg/mL; 95% CI: 27, 316; *P*=0.021) and IL-6 (1321±2200 pg/mL for LPP vs 2604±3039 pg/mL for SPP, MD: 1282 pg/mL; 95% CI: 59, 2505; *P*=0.040). Fold change in expression of HIF1α mRNA between the peritoneal biopsies at the beginning and end of surgery (n=19) is 1.9±0.9 for LPP versus 4.3±3.2 at SPP (MD: 2.3; 95% CI: .04, 4.7; *P*=0.05). Serum levels of HSP70 at the end of surgery are significantly higher at standard pressure (6247±4000 pg/mL) than low pressure (5113±1422 pg/mL) (MD: −1134 pg/mL; 95% CI: 255, 2523; *P*=0.043). Patients who developed infectious complications had a significantly lower ex vivo production capacity of TNFα on POD1 (86±33 pg/mL vs 197±167 pg/mL, MD: 111 pg/mL; 95% CI: 25, 197; *P*<0.001) and TNFα, IL-6, and IL-1β on POD3 (101±51 vs 265±281 pg/mL, MD: 165 pg/mL; 95% CI: 73, 256; *P*<0.001 for TNFα, 2211±1410 vs 3968±3078 pg/mL, MD: 1757 pg/mL; 95% CI: 518, 2997; *P*=0.006 for IL-6, and 468±292 vs 806±654 pg/mL, MD: 338 pg/mL; 95% CI: 73, 603; *P*=0.014 for IL-1β) in comparison to patients without complications (Fig. 3B). In addition, patients with a PACU pain score (NRS) of ≥5 had a significantly lower ex vivo production capacity of TNFα (170±191 vs 396±327 pg/mL, MD: 227 pg/mL; 95% CI: 57, 396; *P*=0.011) and IL-6 (2941±2592 vs 5445±3410 pg/mL, MD: 2503 pg/mL; 95% CI: 679, 4327; *P*=0.009) on POD3 compared with patients with a PACU pain score of 0 to 4. This difference is also present when only considering the patients without complications (196±211 vs 439±361 pg/mL, MD: 243 pg/mL; 95% CI: 62−423; *P*=0.046 for TNFα and 3270±2698 vs 5713±3385 pg/mL, MD: 2443 pg/mL; 95% CI: 439, 4448; *P*=0.018 for IL-6). Patients who developed an infectious complication (n=21) reported significantly higher pain scores in rest at the PACU than patients without complications (n=125) (6.2±2.4 vs 5.0±2.6, MD: 1.2; 95% CI: 0.02, 2.4; *P*=0.046).

**FIGURE 4:**
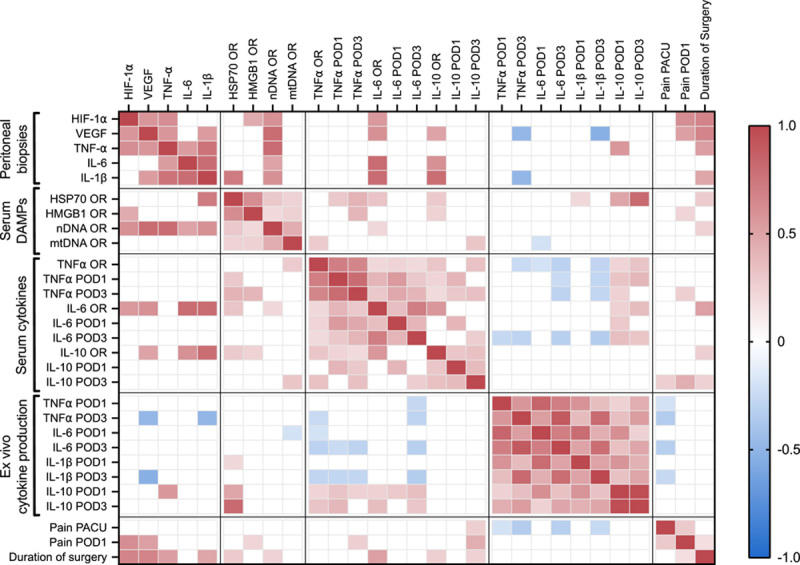
Heatmap of statistically significant correlation coefficients (red for positive correlation, blue for negative correlation) between peritoneal biopsy markers (mRNA level), serum DAMPs, serum cytokines, ex vivo cytokine production capacity, postoperative pain, and duration of surgery. OR indicates operating room.

The correlation matrix in Figure 4 displays the statistically significant correlations (red for positive-, blue for negative correlations) between tissue hypoxia and inflammation markers, serum DAMPs, serum cytokines, ex vivo cytokine production capacity, pain, and duration of surgery. Surgical site markers of hypoxia and inflammation correlate with serum DAMPs (HMGB1, HSP70, and nDNA) and serum cytokines (IL-6 and IL-10). The proinflammatory serum cytokines (TNFα and IL-6) inversely correlate with ex vivo proinflammatory cytokine production capacity (TNFα and IL-6), but positively correlate with ex vivo IL-10 production capacity. Pain scores at the PACU show a negative correlation with ex vivo proinflammatory cytokine production capacity (TNFα, IL-6, and IL-1β).

#### Late Recovery

The questionnaire response rate at 3 months was 83% (148/178). HRQOL quantified with the RAND-36 score 3 months after surgery increased with 3.9±12.4 (scale 0–100) compared with before surgery for LPP, compared with 0.1±10.6 for SPP (*P*=0.047). Quantified with the MPQ, the mean total number of words chosen (MPQ NWC-T) decreases with 0.03±3.7 for SPP versus 1.29±3.1 for LPP (MD: 1.26; 95% CI: 0.15, 2.4; *P*=0.026) from before until 3 months after surgery. The mean total Pain Rating Index (MPQ PRI-T) decreases with −0.01±6.8 for SPP versus 2.31±4.6 for LPP (MD: −2.3; 95% CI: 0.4, 4.2; *P*=0.019) from before until 3 months after surgery (Table 3). RAND-36 score 3 months after surgery shows a moderate negative correlation with the MPQ NWC-T 3 months after surgery (*r*_145_=−0.46, *P*<0.001, and with the MPQ PRI-T at 3 months after surgery (*r*_145_=−0.43, *P*<0.001). QoR-40 on POD1 correlates with MPQ NWC-T (*r*_145_=−0.35, *P*<0.001), MPQ PRI-T (*r*_145_=−0.36, *P*<0.001) and RAND-36 (*r*_145_=0.310, *P*<0.001) at 3 months after surgery.

## DISCUSSION

Our RECOVER trial showed a clear advantage for LPP and deep NMB over SPP and moderate NMB regarding the primary outcome patient-reported quality of recovery (QoR-40) and innate cytokine production capacity from baseline to POD1 after laparoscopic colorectal surgery following the ERAS program. Moreover, patients in the LPP group had lower postoperative pain scores and developed less infectious complications in the first 30 days after surgery. Our results confirm and add evidence to the previously reported benefits for LPP in colorectal laparoscopic surgery regarding quality of recovery,^21^ pain,^5^,^6^ and opioid consumption.^5^ We used the StEP-COMPAC^22^ recommended QoR-40 and found a benefit not on just one domain but for comfort, physical independence, and pain. In contrast to the PAROS trial (median of 4 vs 3 days), no decreased length of stay was observed.^6^ However, the median length of stay in our study was only 3 days in both groups.

Patients operated at LPP showed lower surgical site hypoxia and inflammation markers and circulating DAMPs, with a less impaired early postoperative ex vivo cytokine production capacity. Leijte et al^10^ demonstrated an association between tissue injury, the release of DAMPs, immune suppression, and infectious complications in patients undergoing cytoreductive surgery with hyperthermic intraperitoneal chemotherapy. Our study reveals a similar association in surgical procedures without immune suppression resulting from intraoperative chemotherapy. Furthermore, we demonstrate that decreasing surgical tissue damage can directly abate immune suppression and postoperative infections. The correlation matrix provides a first illustration of the factors presumably involved in the complex interplay between surgical injury and the innate immune response. Starting at the tissue level (parietal peritoneum), we measure an increase in hypoxia (HIF-1α and VEGF) and inflammatory markers (TNFα, IL-1β, and IL-6) at mRNA level between the biopsies at the beginning and end of laparoscopy. Hypoxia-inducible factors are transcription factors that, under normal physiological conditions, are degraded by propyl hydroxylases that require oxygen as a cofactor (reviewed in the study by Yuan et al^23^). In the case of hypoxia, HIF-1α is not degraded but stabilized and migrates into the nucleus to regulate the transcription of genes controlling metabolism, inflammation, apoptosis, and angiogenesis.^22^ As hypothesized, the increase in HIF-1α mRNA in peritoneal biopsies is more than twice as high for SPP at the end of the surgery, implicating a higher level of hypoxia-reperfusion injury. HIF-1α in turn regulates the expression of VEGF.^24^ These local tissue markers and tissue cytokines correlate with serum DAMPs (HMGB1 and nDNA) and serum cytokines (IL-6 and IL-10), indicating the spread of tissue damage molecules into the circulation followed by a systemic innate immune response. DAMPs are known to bind to toll-like receptors and induce proinflammatory cytokines.^25^ Surgical injury-induced inflammation is normally followed by a protective compensatory postoperative anti-inflammatory phenotype, where more extensive injury may even induce immune paralysis.^26^ This mechanism is directly illustrated by the correlation of proinflammatory serum cytokines TNFα and IL-6 with ex vivo production capacity of the anti-inflammatory IL-10 and the inverse correlation with ex vivo production capacity of TNFα, IL-6, and IL-1β. Ex vivo cytokine production capacity upon endotoxin stimulation is a dynamic and relevant measure as it represents the ability of the innate immune cells to respond when challenged by a pathogen. We show that LPP leads to less tissue hypoxia, lower circulating tissue damage markers (HSP70) resulting in a less impaired postoperative innate cytokine production capacity. Patients undergoing colorectal surgery are eminently at risk for infections due to the combination of a by default contaminated surgical area, underlying diagnoses and exposure to many factors that impair wound healing.^27^ Decreasing tissue injury and maintaining immune homeostasis in the RECOVER study resulted in a 10% reduction in postoperative infections. In addition, we again confirm the previously described strong association between early postoperative pain and infectious complications.^28^,^29^ It seems compelling that surgical injury and DAMPs are the predominant common precursors for pain and immune modulation. Conjointly, surgical injury may cause pain and the resulting stress response may influence immune homeostasis. It is well established that the innate immune response plays a crucial role in antitumor activity to prevent tumor progression and metastases,^30^,^31^ which adds to the importance of preventing immune suppression in this population. To our knowledge, only 1 observational study previously demonstrated that increased plasma levels of DAMPs are associated with a decreased ex vivo production capacity and infectious complications after hyperthermic intraperitoneal chemotherapy surgery,^8^ but this study did not have sufficient power to detect a correlation between ex vivo cytokine production capacity upon endotoxin stimulation and infectious complications. Our study established this correlation and is also the first to demonstrate that a specific intervention that decreases surgical tissue injury and circulating DAMPs (HSP70), lowering IAP, results in the preservation of innate immune homeostasis and less infectious complications after surgery.

Major prerequisites of adapting a surgical technique are safety and feasibility. Our finding that surgery could safely be completed at LPP in 75% of patients with the same duration of surgery is consistent with a reported 75% to 83% reported in previous trials.^4^,^5^ IPP-ColLapSe II even reports less intraoperative events for low IAP laparoscopy. Deep NMB is an important facilitator for low IAP applied in all these trials, as with LPP facilitated by moderate NMB more intraoperative events have been reported.^32^ Maintaining deep NMB throughout surgery might be a challenge in clinical practice, as adequate titration of rocuronium to reach the small range of PTC 1 to 2 requires continuous quantitative neuromuscular monitoring and dosage adjustments. Second, many anesthesiologists associate deep NMB with an increased risk of postoperative pulmonary complications. However, this often is the result of inadequate neuromuscular monitoring and reversal of NMB (reviewed in the study by Nemes et al^33^). In one of the most prominent recent trials on this topic, the POPULAR trial,^34^ only 16.5% of 17,150 patients were monitored and extubated according to the international consensus guideline.^35^ Therefore, when using neuromuscular blocking agents close monitoring is mandatory. As mentioned in the introduction, studies investigating only the effects of deep NMB find little to no effect on postoperative pain and quality of recovery on POD1, indicating the reported clinical benefits can predominantly be attributed to low pressure.^7^,^8^,^36^

The additional value of our trial consists not only of new insights into the relationship between perioperative innate immune function and clinical outcomes but also provides the first data on long(er)-term effects of surgical injury and immune homeostasis on chronic pain and HRQOL 3 months after surgery. As previously shown for laparoscopic donor nephrectomy,^37^ the relationship between acute pain, chronic pain, and long-term HRQOL is also present for laparoscopic colorectal surgery. Nonetheless, while statistically significant, the clinical relevance of the difference for MPQ number of words chosen and pain rating index can be questioned. A major strength of our study was the accuracy of the intervention. For deep NMB, the target PTC of 1 to 2 was reached for 73% of all 5-minute measurements. Moreover, we prospectively collected all ERAS criteria and both groups showed the same high percentage of adherence. A possible limitation of using the total QoR-40 as the primary outcome is that the domains support and emotions appear uninfluenced by IAP and NMB. Still, the emotional state of the patient (eg, feeling anxious or sad) may very well be influenced by or represent the postoperative physical hindrances like pain and nausea, and therefore be reflected in both domains. In our study, there was no statistically significant difference in perceived support or emotions between the groups. Other limitations of the study were that the individual length of hospital stay may have led to bias, if patients were discharged before the third POD, the POD3 blood samples and pain scores were missing. This was not the case for the questionnaires, as they were taken home and returned by regular post. Missing data due to loss to follow-up may have affected the late recovery outcomes after 3 months, however, given the relatively high response rate of 83%, this influence is likely limited. Second, the methodology of the substudy was not included in the prepublished protocol, however, it was preplanned, approved by the medical ethical committee, and concisely published at clinicaltrials.gov. For the substudy, we chose to show the intention-to-treat and per-protocol analysis, as the per-protocol analysis may most closely reflect the underlying scientific model,^38^ which is primarily of interest to illustrate the relationship between damage from IAP and the ensuing systemic immune response. Granted, a per-protocol analysis may introduce substantial bias and results need to be interpreted with caution. Last, the study experienced a delay due to the coronavirus disease 2019 pandemic, and coronavirus disease 2019 isolation measures may also have affected the quality of life and recovery. Nonetheless, no statistically significant differences were observed between outcomes before and during the pandemic.

While safe and attainable, the early and long-term advantages of LPP during colorectal laparoscopic surgery are very compelling and LPP facilitated by deep NMB would be a valuable addition to the intraoperative elements of the future colorectal ERAS program.
